# Agreement between Orbscan II, VuMAX UBM and Artemis-2 very-high frequency ultrasound scanner for measurement of anterior chamber depth

**DOI:** 10.1186/1471-2415-14-20

**Published:** 2014-02-25

**Authors:** Haya Matuoq Al Farhan

**Affiliations:** 1 Department of Optometry and Vision Sciences, College of Applied Medicine Sciences, King Saud University, P.O. Box 10219, Riyadh 11433, Kingdom of Saudi Arabia

**Keywords:** Anterior chamber depth, Artemis-2 VHF, Normal eyes, Orbscan II, Ultrasound biomicroscopy, Ultrasound scanner

## Abstract

**Background:**

The aim was to compare the anterior chamber depth (ACD) measurements taken with Orbscan II, ultrasound biomicroscopy (UBM) and the Artemis-2 VHF (very-high-frequency) ultrasound scanner in normal subjects.

**Methods:**

In this prospective study, one eye from each of 60 normal subjects was randomly selected. Three subjects dropped out of the study because they were apprehensive about the UBM examination; their data were excluded entirely. Measurements of ACD were taken with the Orbscan II, UBM and Artemis-2 VHFUS. Results were obtained for coefficient of variance (CV) and intra-class correlation coefficient (ICC), and statistical analysis was by repeated-measures analysis of variance (ANOVA) for intra-observer repeatability. ANOVA and Bland–Altman analyses were used to determine limits of agreement (LOA) between the three instruments.

**Results:**

The average ACD (± standard deviation) was 3.13 ± 0.34 mm, 2.96 ± 0.27 mm and 2.87 ± 0.31 mm for the Orbscan II, UBM and Artemis-2 VHFUS, respectively. The repeatability scores were 0.015 ± 0.014%, 0.08 ± 0.09% and 0.07 ± 0.06% for the Orbscan II, UBM and Artemis-2 VHFUS, respectively. The ICC for repeatability of Orbscan II, UBM and Artemis-2 VHFUS measurements was high and equal to 0.99%. The intra-observer repeatability scores of the ACD measurement p-values using Orbscan II, UBM and Artemis-2 VHFUS were 0.12, 0.70 and 0.10, respectively. The mean difference and standard deviations for ACD measurements using Orbscan II *vs* UBM, Orbscan II *vs* Artemis-2 VHFUS and UBM *vs* Artemis-2 VHFUS were 0.17 ± 0.31 mm, 0.27 ± 0.34 mm and 0.10 ± 0.18 mm, respectively. LOAs were 0.78 to -0.44 mm, 0.93 to -0.39 mm and 0.45 to -0.26 mm. ANOVA revealed a statistically significant difference between the Orbscan II, UBM and Artemis-2 VHFUS (p < 0.0001).

**Conclusions:**

Measurements by the three instruments show high repeatability. UBM and the Artemis-2 VHFUS can be used interchangeably, but the Orbscan II cannot be used interchangeably with UBM or the Artemis-2 VHFUS.

## Background

It is well-documented that anterior chamber depth (ACD) measurement is essential as a screening method for primary angle-closure glaucoma [1,2]; it is also used in biometry to calculate the power of intraocular lenses (IOLs) [3], for determining the precise optic zone ablation diameter in keratorefractive surgery [4], and in postoperative assessments [5].

Recently, new techniques have been introduced to assess ACD using either ultrasonic or optical technologies [6]. Ultrasonic immersion instruments include the ultrasound biomicroscope (UBM; Sonomed Inc., New York, USA) [7-9] and the Artemis-2 VHFUS scanner (Scott Philips Engineering, Victoria, BC, Canada) [10]. Optical methods that have been studied include anterior segment optical coherence tomography (OCT) [11] and Scheimpflug imaging [12].

Ultrasound and optical techniques allow quantitative measurements of ACD. Optical methods are objective, however it is difficult to accurately measure ACD in the presence of edema, opacities, scarring or deposits in the optical media [12]. In contrast, ultrasound-based methods do not require clear optical media to obtain precise measurements of ACD. There are disadvantages of UBM: for example, the examiner must manually adjust the transducer head to maximize centrality and perpendicularity of the image, which takes time, and analog-based UBM does not image the interface consistently because analog processing does not produce a high enough signal-to-noise ratio between the interface echo complex and the surrounding tissue [13].

Previous studies have investigated the agreement between ACD measurements obtained with these different instruments [7-13] but, to the best of our knowledge, this is the first study to compare measurements obtained with these devices in normal eyes. The aim of this study was to assess the repeatability and agreement of ACD measurements obtained using the Orbscan II topography system, UBM and the Artemis-2 VHFUS.

## Methods

The study enrolled 60 consecutive, healthy, oculovisually normal subjects (33 right eyes and 27 left eyes) aged 19–30 years (22 ± 2 years). Comprehensive anterior segment examinations of all eyes were performed using a slit lamp. The exclusion criteria were: history of any intraocular or corneal surgery; contact lens wear; systemic diseases such diabetes mellitus; intraocular pressure (IOP) of 20 mmHg or more; corneal anomalies; spherical refraction of 4.00 diopters (D) or more; or cylindrical refraction of 2.00 D or more [10]. Spherical and cylindrical refractions and IOPs were determined by autorefractometry (Auto Kerato-Refracto-Tonometer TRK-1P-Topcon, Inc., Tokyo, Japan).

One eye was randomly selected in each subject using a table generated on Microsoft < tm > Excel. All measurements were obtained in the afternoon and between 12:00 pm and 3:00 pm[14] by a single investigator, in the same clinic at one location, under mesopic conditions [13]. ACD was defined as the measured distance from the corneal endothelium to the anterior lens surface.

Measurements were first taken using the non-invasive Orbscan II technique, and followed by the Artemis-2 VHFUS. This sequence was intended to control for any variations in central corneal thickness caused by changes in the resistance of the cornea from indentation [15]. Eyes were then rested for 1 hour, after which UBM was used; this rest period was avoided any inadvertent corneal indentation errors during UBM [16]. Three repeated measurements were obtained consecutively by each method, for each eye. These measurements were then compared to obtain repeatability scores for each instrument and agreement between the instruments. Three subjects dropped out of the study when they became apprehensive about the UBM examination. All of their associated data were excluded from the analysis.

The purpose of the study was explained to all subjects and informed consent was obtained from each before the examination began. The study conformed with the ethical considerations laid out in the 2008 Declaration of Helsinki and the study protocol was approved by the research ethics review board of the College of Applied Medicine Science at King Saud University.

### Orbscan II method

The Orbscan II (Bausch & Lomb, Rochester, NY, USA) is a non-contact optical computerized slit-scanning topography system. It quantifies elevation differences between the anterior and posterior corneal surfaces, the anterior surfaces of the iris and the lens, and automatically compensates for differences in refraction from the corneal endothelial surface using a ray-trace algorithm, to provide accurate and reproducible measurements of ACD from both surfaces of the cornea to the anterior surface of the crystalline lens along the optical axis. Each subject was seated in a typical normal position using the chin-rest; the instrument was aligned and scans were made of the cornea. The system software then automatically detected the corneal endothelial surface and anterior surface of the crystalline lens on the acquired images, compensating for differences in refraction from the corneal anterior surface using a ray-trace algorithm, and calculating ACD [17].

### Artemis-2 VHFUS method

The Artemis-2 VHFUS (Scott Philips Engineering, Victoria, BC, Canada) has an advantage over other instruments in that during scanning, the probe is moved in an arc-shaped trajectory which is matched approximately to the corneal curvature, enabling near-normal incidence at all positions. The device incorporates a fixation light and optical camera for visualization of the eye to assure centration [18]. With the subject in the seated position, and his or her face on a three-point forehead and chin-rest, the eye is placed into a soft-rimmed eye-cup similar to that in swimming goggles. The compartment in front of the eye was filled with a sterile coupling fluid filled and scanning was performed via an ultrasonically transparent (sterile) membrane, without the need for a speculum. Thus, there was no contact between the scanner probe and the eye. The 3-D scan took 2–3 minutes for each eye.

### UBM method

The UBM is an immersion technique that uses a high-frequency (50 MHz) ultrasound beam to measure various ocular parameters. The examiner manually adjusts the transducer head to maximize the centrality and perpendicularity of the images, which can be a time-consuming process. The VuMAX™ UBM (Sonomed Inc., NY, USA) was used. First, one drop of topical anesthesia (0.4% benoxinate hydrochloride) was instilled in the subject’s eye. The eye-cup was disinfected with an alcohol swab and filled with a 1% methylcellulose solution. The transducer head was immersed in this solution and the eye cup was placed on the sclera of the eye. The subject was then asked to look at a fixation target on the ceiling in order to maintain steady accommodation and fixation while the image was obtained.

### Statistical methods

The data for all subjects were analyzed using Microsoft Excel 2007 < tm>. For data analysis, Medcalc software (version 11.4.4.0) was used. Coefficient of variance (CV) was used to show the extent of variability in relation to mean of the population. The intra-class correlation coefficient (ICC) was used to assess inter-rater reliability. Repeated-measures analysis of variance (ANOVA) was used to test the statistical significance of the repeatability of three intra-observer readings, and of the mean of the three readings from the three instruments. Bland–Altman analyses were applied to determine limits of agreement (LOA) between the instruments. All tests were two-tailed. The level of statistical significance for this study was set at 0.05.

## Results

The study included 60 normal eyes. The mean spherical equivalent refractive error ± standard deviation (SD) was -0.50 ± 1.00 D. The mean IOP (± SD) was 14.00 ± 2 mmHg. The mean ACD (± SD) measurements using Orbscan II, UBM and Artemis-2 VHFUS were 3.13 ± 0.34 mm, 2.96 ± 0.27 mm and 2.87 ± 0.31 mm, respectively.

### Intra-observer repeatability of ACD measurements

The intra-observer repeatability of ACD measurements with Orbscan II, UBM and Artemis-2 VHFUS are summarized in Table 1. The intra-session reproducibility of ACD measurements was excellent for all three instruments as shown by the high ICCs, which are shown in Table 1.

**Table 1:** The intra-observer coefficient of variance (CV) (%) in anterior chamber depth (ACD) measurements and intra-session repeatability for Orbscan II, Artemis-2 and UBM evaluated by intra-class correlation coefficient (ICC)

	**Orbscan II**	**Artemis 2**	**UBM**
CV mean ± SD (%)	0.015 ± 0.014	0.08 ± 0.09	0.07 ± 0.06
Significance of differences between CVs (p value)	0.12	0.10	0.70
ICC (95% CI)	0.990 (0.984–0.994)	0.990 (0.984–0.994)	0.990 (0.984–0.994)

CI, confidence interval; SD, standard deviation; UBM, ultrasound biomicroscopy.

**Table 2:** The mean differences ± standard deviation (SD), limits of agreement and pair-wise comparisons of p-values for anterior chamber depth (ACD) measurements using the three instruments

	**Mean difference ± SD**	**Limits of agreement**	p-values*
Orbscan II *vs* UBM	0.17 ± 0.31 mm	0.80 to - 0.43 mm	*<* 0.01
Orbscan II *vs* Artemis-2 VHFUS	0.27 ± 0.34 mm	0.94 to - 0.39 mm	*<* 0.001
UBM *vs* Artemis-2 VHFUS	0.10 ± 0.18 mm	0.45 to - 0.26 mm	*>* 0.05

UBM, ultrasound biomicroscopy; VHFUS, very high-frequency ultrasound scanner. *Pairwise comparison.

### Agreement between the three instruments

**Figure 1:**
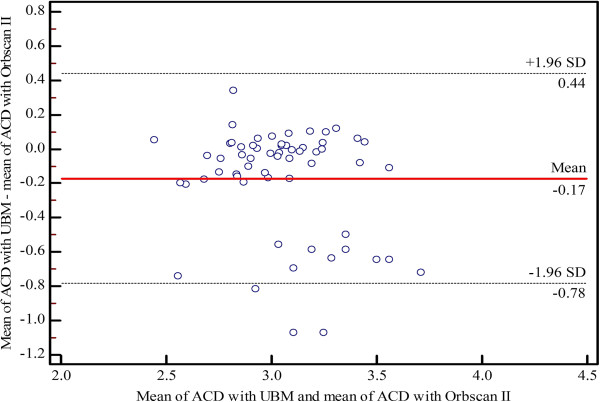
Bland–Altman plot of agreement showing the mean differences and limits of agreement of anterior chamber depth (ACD) measurements between UBM and Orbscan II.

**Figure 2:**
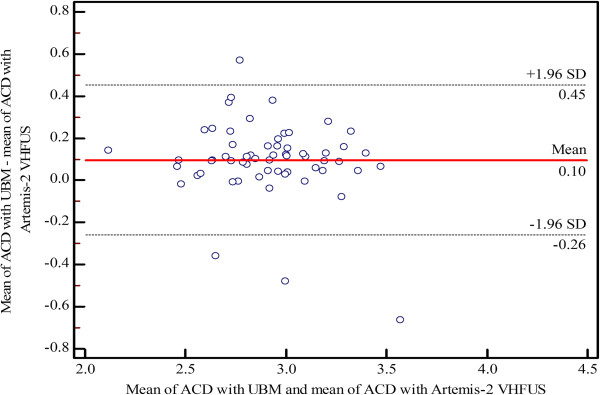
Bland–Altman plot of agreement showing the mean differences and limits of agreement of anterior chamber depth (ACD) measurements between UBM and Artemis-2 VHFUS.

**Figure 3:**
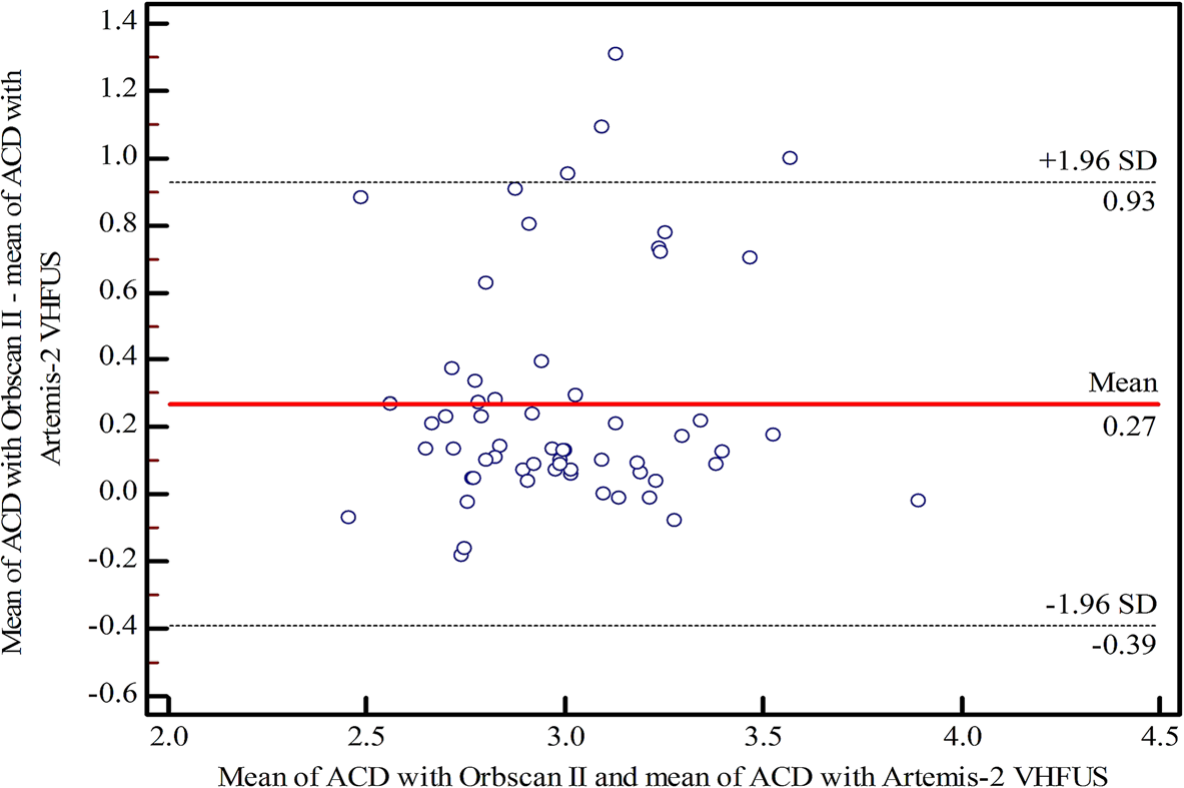
Bland–Altman plot of agreement showing the mean differences and limits of agreement of anterior chamber depth (ACD) measurements between Orbscan II and Artemis-2 VHFUS.

The mean differences, SDs, LOA and pairwise comparisons (using p-values) for ACD measurements using the Orbscan II, UBM and Artemis-2 VHFUS are summarized in Table 2. There was a statistically significant difference between mean ACD measured with the Orbscan II, UBM and Artemis-2 VHFUS, as determined by ANOVA (p = 0.0001). Pairwise comparisons of the Orbscan II *vs* UBM and Orbscan II *vs* Artemis-2 VHFUS showed a statistically significant difference (p <0.01 and p < 0.001, respectively), but there was no statistically significant difference found for UBM *vs* Artemis-2 VHFUS (p > 0.05; Table 2). The mean difference percentage of ACD measurements for UBM and Artemis-2 VHFUS was 3.1%. Bland–Altman plots of the mean differences and LOA of ACD measurements using the three instruments are shown in Figures 1, 2 and 3, respectively).

## Discussion

An accurate ACD measurement is required to prevent endothelial damage during cataract surgery [19] and in cases of IOL complications that require removal or exchange, such as unexpected or unintended refractive outcomes caused by biometry errors [20]. Measurement of ACD is also a common technique in pseudophakic eyes, for evaluating the possibility of IOL movement during accommodation [21]. Additionally, several studies report that ACD can be used to predict glaucoma, whereby a shallow ACD increases the risk of developing acute and chronic angle-closure glaucoma [1,2].

In the present study, the intra-observer repeatability of ACD measurements using the Orbscan II, UBM and Artemis-2 VHFUS was not significantly different (p ≤ 0.05). The CVs and ICCs demonstrated high levels of consistency and repeatability for the three instruments. The Bland–Altman analysis of the ACD measurements showed poor LOA between the Orbscan II *vs* UBM and the Artemis-2 VHFUS, respectively. However, there was a high level of agreement between the UBM and the Artemis-2 VHFUS. The mean difference was 0.09 mm, which is insufficient to affect decisions related to refractive surgery in clinical practice. The ANOVA indicated a statistically significant difference between Orbscan II, UBM and Artemis-2 VHFUS for ACD measurements (p = 0.0001), although the p-values of the pairwise comparisons of mean ACD measurements by Orbscan II *vs* UBM, Orbscan II *vs* Artemis-2 VHFUS, and UBM *vs* Artemis-2 VHFUS were 0.01, 0.001 and 0.05, respectively. The mean ACD measured with Orbscan II *vs* UBM and Artemis-2 VHFUS was significantly different; this demonstrates that these instruments cannot be used interchangeably. However, mean ACD measurements using UBM and Artemis-2 VHFUS were not significantly different and, therefore, these instruments can be used interchangeably. Moreover, the mean difference percentage for ACD measurements was 3.1%, which further indicates that ACD measurements using UBM and Artemis-2 VHFUS are equally valid.

A number of studies have reported high intra-observer repeatability of ACD measurements obtained using Orbscan II, UBM and Artemis-2 VHFUS in normal eyes [8,9,11,22]. Our results show high intra-observer consistency and repeatability of ACD measurements obtained with all three instruments.

Previous studies comparing ACD measurements obtained by optical and contact ultrasonic instruments reported small differences between different optical techniques (< 5%), yet larger differences (10–15%) have been reported with different contact ultrasonic techniques [23,24]. Furthermore, ACD measurements made by contact ultrasonic techniques are consistently about 0.40 mm smaller than those measured using optical techniques, such as Orbscan and anterior-segment OCT [9,23], and about 0.30 mm less than those obtained with immersion ultrasound [24]; this might be due to the mechanical applanation effect that occurs when the ultrasound probe touches the cornea. Our results show that the difference (expressed as a percentage) for UBM *vs* Artemis-2 VHFUS was 3.1%, which is comparable to that previously reported for optical techniques. The mean differences of the ACD measurements using UBM and the Artemis-2 VHFUS were less than those for the Orbscan II – by 0.18 mm and 0.27 mm, respectively. This is probably because the ultrasonic instruments used in this study employ immersion techniques that reduce any mechanical applanation effect.

Some studies have reported that ACD measurements change according to the accommodative status of the eye [25,26]. For instance, in a comparison of Orbscan II *vs* anterior segment OCT or Scheimpflug camera in normal healthy subjects (mean age 23.3–25 years), mean ACD measurements ranged from 0.10–0.15 mm and 0.08–0.23 mm, respectively [11,15,27]. This might be because Orbscan II and Scheimpflug cameras do not have fixation systems to block accommodation of the eye, unlike anterior segment OCT, which incorporates a fixation light and optical camera for visualization of the eye to assure centration [4,10,15]. The Artemis-2 VHFUS used in this study incorporates both fixation light and optical camera for visualizing the eye and ensuring centration. During the UBM examination, subjects were instructed to fixate on a ceiling target to maintain accommodation. During the scanning session, the examiner looked for any decentration or loss of fixation [28]. Our results show there were no statistically significant differences between UBM and Artemis-2 VHFUS, most likely due to similarity between the techniques and constant fixation.

Previous studies have shown variations in the mean ACD value obtained by different instruments, which may be due to differences in sample size, study population [7,23,29,30] and refractive error [7,23,29,31]. Table 3 summarizes the results from three recent studies looking at the repeatability of different instruments for measuring ACD. Lee *et al.*[7] reported that ACD measurements (mean ± SD) from Orbscan II and UBM were 2.82 ± 0.46 mm and 2.91 ± 0.43 mm, respectively. The mean difference was 0.09 ± 0.09 mm and the p-value was 0.001, indicating that instruments cannot be used interchangeably [7]. Our results also show that the Orbscan II, UBM and Artemis-2 VHFUS cannot be used interchangeably. However, the mean differences of the ACD measurements of Orbscan II *vs* UBM and Orbscan II *vs* Artemis-2 VHFUS in our study were slightly higher than their results, possibly because of differences in the subjects; their study was on older subjects (mean age 44.25 years) and some had cataracts [29]. Although there was just one observer in this study, which may be considered as a potential limitation, there was a high degree of intra-observer agreement.

**Table 3:** Overview of recent studies comparing the repeatability of different instruments for measuring anterior chamber depth (ACD)

**Author (year)**	**Instruments**	**Parameter**	**Mean age ± SD**	**Spherical equivalent range**	**Agreement between results?**^**‡**^
Lee *et al.* (2007) [7]	Orbscan II* *vs* UBM^**^	ACD	44.25	-5.50 to + 3.00 D	No
Hashemi *et al.* (2005) [30]	Orbscan I* *vs* USP^†^	ACD	30.2 ± 8.5	-15.00 to - 1.00 D	No
Chaidaroon and Jengialern (2005) [31]	Orbscan I* *vs* IOL Master*	ACD	27.90 ± 8.80	-8.49 ± 4.00 D	No

SD, standard deviation; UBM, ultrasound biomicroscopy; USP, ultrasound probe.

*Optical non-contact.

**Ultrasound immersion.

^†^Ultrasound contact.

^‡^In all studies the difference between instruments was statistically significant.

## Conclusions

All three instruments in this study demonstrated high repeatability. In terms of obtaining measurements of ACD, the Orbscan II cannot be used interchangeably with UBM or Artemis-2 VHFUS, but the UBM and Artemis-2 VHFUS can be used interchangeably for the measurement of ACD.

## Competing interests

The author declares that she has no competing interests.

## Author’s contributions

The author was solely responsible for the study’s conception and design, for the analysis and interpretation of data, and for drafting and revising the manuscript critically for important intellectual content.

## Pre-publication history

The pre-publication history for this paper can be accessed here:

http://www.biomedcentral.com/1471-2415/14/20/prepub
